# Allergic inflammation alters the lung microbiome and hinders synergistic co-infection with H1N1 influenza virus and *Streptococcus pneumoniae* in C57BL/6 mice

**DOI:** 10.1038/s41598-019-55712-8

**Published:** 2019-12-18

**Authors:** Kim S. LeMessurier, Amy R. Iverson, Ti-Cheng Chang, Maneesha Palipane, Peter Vogel, Jason W. Rosch, Amali E. Samarasinghe

**Affiliations:** 1 Department of Paediatrics, College of Medicine, University of Tennessee Health Science Center, Memphis, TN 38103 USA; 2 Children’s Foundation Research Institute, Memphis, TN 38103 USA; 3 Department of Infectious Diseases, St. Jude Children’s Research Hospital, Memphis, TN 38105 USA; 4 Center for Applied Bioinformatics, St. Jude Children’s Research Hospital, Memphis, TN 38105 USA; 5 Department of Veterinary Pathology at St. Jude Children’s Research Hospital, Memphis, TN 38105 USA

**Keywords:** Microbiome, Influenza virus, Mucosal immunology

## Abstract

Asthma is a chronic airways condition that can be exacerbated during respiratory infections. Our previous work, together with epidemiologic findings that asthmatics were less likely to suffer from severe influenza during the 2009 pandemic, suggest that additional complications of influenza such as increased susceptibility to bacterial superinfection, may be mitigated in allergic hosts. To test this hypothesis, we developed a murine model of ‘triple-disease’ in which mice rendered allergic to *Aspergillus fumigatus* were co-infected with influenza A virus and *Streptococcus pneumoniae* seven days apart. Significant alterations to known synergistic effects of co-infection were noted in the allergic mice including reduced morbidity and mortality, bacterial burden, maintenance of alveolar macrophages, and reduced lung inflammation and damage. The lung microbiome of allergic mice differed from that of non-allergic mice during co-infection and antibiotic-induced perturbation to the microbiome rendered allergic animals susceptible to severe morbidity. Our data suggest that responses to co-infection in allergic hosts likely depends on the immune and microbiome states and that antibiotics should be used with caution in individuals with underlying chronic lung disease.

## Introduction

Lung diseases are a leading cause of morbidity and mortality worldwide. Acute and chronic respiratory diseases, excluding infections, affect greater than 12% of the population in the United States and hundreds of millions worldwide^1^. Asthma is the most prevalent of these^2^ and has the greatest economic burden^3^, in addition to being one of most challenging lung conditions to investigate and treat. While the exact etiology of asthma remains unstipulated, specified endotypes based on symptoms, immunologic profiles, genes, and environment, are confounded by gender and age. Furthermore, asthma exacerbations can be triggered by respiratory viral infections^4,5^, and some reports suggest that asthmatics are at risk for bacterial pneumonia^6,7^.

Over four million deaths every year (predominantly in children <5 years) are caused by acute respiratory infections^8^. Influenza and pneumococcal disease contribute to approximately one million hospitalizations annually in the U.S^9,10^. While influenza alone can be fatal, recovering patients have increased susceptibility to respiratory bacterial infections^11^, of which *Streptococcus pneumoniae* (*Spn*), a pathogen associated with community-acquired pneumonia^12^, is highly associated with severe disease and is a cause for excess mortality during influenza^13^. The ‘Spanish Flu’ pandemic exemplified this predilection with the majority of deaths attributed to subsequent bacterial infections^14^, as did the 2009 Swine Flu pandemic in which 29–55% of deaths resulted from secondary bacterial pneumonia^15,16^. Approximately 25% of hospitalized patients during the Swine Flu pandemic had asthma^17^ indicating that these three disease conditions may have high overlap during influenza seasons. Furthermore, the occurrence of pulmonary infections in asthmatics is augmented by high disease incidences of each, and seasonal overlap between infectious agents and allergens.

Although asthma was a risk factor for hospitalization during the 2009 ‘Swine Flu’ pandemic^18^, subsequent studies noted that asthmatics had less severe outcome (including reduced bacterial pneumonia) compared to non-asthmatics^17,19–22^. Explanations for this unexpected and counterintuitive outcome are sparse, although possibilities include steroid use^23^, heightened medical care, and increased likelihood of vaccinations in asthmatics^17^. As noted in our recent meta-analysis^17^, most cohorts had patients that had multiple underlying chronic diseases. Since hospitalized asthmatics were less likely to suffer severe morbidities associated with IAV infection such as increased length of stay, intensive care unit admission, requirement for mechanical ventilation, and overall, were less likely to die from influenza compared to non-asthmatics^17^, there is clinical precedence that the disease pathogenesis of influenza in asthmatics is conceivably different especially during the asthma exacerbation induced by the virus. Mechanisms associated with this phenomenon are likely to be multifaceted as indicated by animal models^17^. Exact mechanisms and outcomes in humans are not well established in humans due to the inaccessibility of mucosal tissue samples limiting thorough mechanistic interrogations. Therefore, animal model systems are invaluable in such instances, and yet, those that allow examination into the convergence of immunologically distinct conditions like asthma, influenza, and pneumococcal pneumonia are lacking.

Effective interrogation of host-pathogen interactions during allergic asthma and respiratory infections necessitates a single animal model that can capture the nuances of morbidities that are immunologically distinct. Recent work with our animal model of asthma and influenza comorbidity underscored the importance of the state of the allergic airways at the time of viral infection in the pathogenesis of influenza in allergic hosts^24^, and identified a novel antiviral function for eosinophils^25^. Although viral-bacterial synergy has previously been established to cause severe pneumonia and mortality^26^, studies that investigated the effect of an allergic microenvironment in the lung on subsequent co-infections with IAV and *Spn* were hindered due to the absence of an effective experimental model. In order to fill this critical gap in technology, we developed and characterized a mouse model of asthma, influenza, and pneumococcal pneumonia (‘triple-disease’) through the combination of our fungal asthma model^27^ with a well-employed model of IAV and *Spn* co-infection^28^ thereby enabling the investigation of host-pathogen interactions in the *in vivo* setting. Our findings suggest that pre-existing allergic asthma protects the host from severe morbidity, as shown by maintenance of weight, and reduced viral-bacterial synergism. Allergic mice also had reduced bacterial burdens, altered inflammatory cell profiles (more eosinophils and macrophages and fewer neutrophils) as well as a distinct lung microbiome compared to those with IAV and *Spn* co-infection alone. Inducing dysbiosis with antibiotics caused a partial reversal of this protective phenotype observed in the allergic mice.

## Results

### Allergic airways inflammation protected mice against severe disease from co-infection

**Figure 1:**
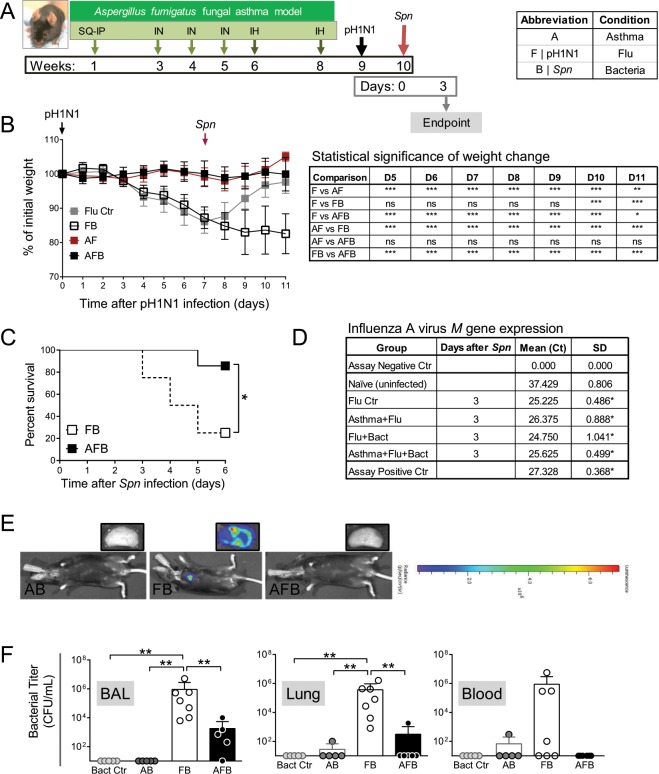
Synergistic morbidity from influenza and bacterial pneumonia are reduced in animals with allergic asthma. Timeline of triple-disease model (**A**) wherein allergen sensitized and challenged mice are infected with influenza A virus (pH1N1) and *Streptococcus pneumoniae* (*Spn*). Weight loss in each group and comparative statistics (two-way ANOVA with Sidak’s multiple comparisons test) associated with weight loss (**B**). Survival curves of FB compared to AFB analysed by log-rank test (**C**). Influenza A virus *M* gene expression in mice 3 days after *Spn* infection compared to uninfected naïve mice analysed by one-way ANOVA with Dunn’s multiple comparisons test (**D**). Bioluminescence imaging for bacteria in mice and harvested lung lobes (**E**). Conventionally measured bacterial load in the bronchoalveolar lavage (BAL), lung homogenate, and blood in each group infected with *Spn* analysed by one-way ANOVA with Dunn’s multiple comparisons test (**F**). Data are representative of one study from four independent studies collected at 3 days post *Spn* infection. n = 5–7 mice in each group. *P < 0.05, **P < 0.01 and ***P < 0.001. SQ: subcutaneous; IN: intranasal; IH: inhalation.

Mouse model systems that can simulate complex interactions between asthma and respiratory infections are limited, but important to study disease-disease interactions that may alter host responses. Since respiratory infections with viruses and bacteria are considered triggers for the development of asthma, infectious agents were utilized prior to allergen provocation^29^. However, while asthma can indeed be triggered by respiratory infections, it can also be exacerbated by the same^30,31^. Herein, our goal was to develop and characterize a model system in which respiratory infections occurred in established allergic airways disease. Mice were subjected to *Aspergillus fumigatus* allergen sensitization and challenge^27,32^, infected with IAV one week after the second fungal challenge^24^, and infected with *S. pneumoniae* seven days later (Fig. 1A). A ubiquitous and clinically relevant fungal allergen^32^ was chosen to mimic the human disease as nearly 70% of patients with severe asthma have fungal sensitizations^33^ with *Aspergillus* species being dominant^34^. Naïve mice were used to measure baseline, while asthma-only, influenza-only (Flu Ctr), bacteria-only (Bact Ctr) mice served as single disease controls. Dual condition groups included Asthma + Flu (AF), Asthma + Bact (AB), and Flu + Bact (FB), while Asthma + Flu + Bact (AFB) triple-disease condition served as the experimental group.

As previously demonstrated by us^24^, IAV infection during peak airways inflammation did not induce weight loss in allergic mice (AF group, Fig. 1B), whereas the same dose of virus triggered about a 12% weight loss in non-allergic mice (F group, Fig. 1B). Non-allergic mice that were co-infected (FB group) lost ~20% weight at the termination point in this study (Fig. 1B), continued to lose weight and succumbed to disease by 6 dpi with *Spn* (Fig. 1C). In stark contrast, allergic mice that were subsequently co-infected (AFB group) did not lose weight and had a comparable weight profile to the AF group (Fig. 1B), and >85% in the AFB group survived compared to 25% in the FB group by day 6 after *Spn* (Fig. 1C). As such, although our primary interest was in the immune responses during disease morbidity, allergic asthma appeared to delay/protect mice from IAV + *Spn*-induced mortality which may provide a time advantage for clinical therapeutic intervention in asthmatics. Infectious virus was absent in the lungs of mice in all groups at 3 days post *Spn* (data not shown) which differs from previous studies that have demonstrated a viral rebound after *Spn* co-infection, albeit using the laboratory strain of IAV^35^. However, measurement of the viral *M* gene expression showed that similar levels of viral gene product existed between groups at 3  days after *Spn* (Fig. 1D). The bacterial burden in the allergic lungs was not sufficient to visualize by fluorescence like in co-infection alone (Fig. 1E), but conventional enumeration of pneumococci on blood agar showed significantly reduced loads in allergic mice compared to the non-allergic co-infected mice (Fig. 1F). Bacterial dissemination into the blood may also be delayed/reduced in allergic mice (Fig. 1F).

### Allergic mice had a more diverse immune cell signature in the airways although tissue inflammation was lower compared to non-allergic mice during co-infection

**Figure 2:**
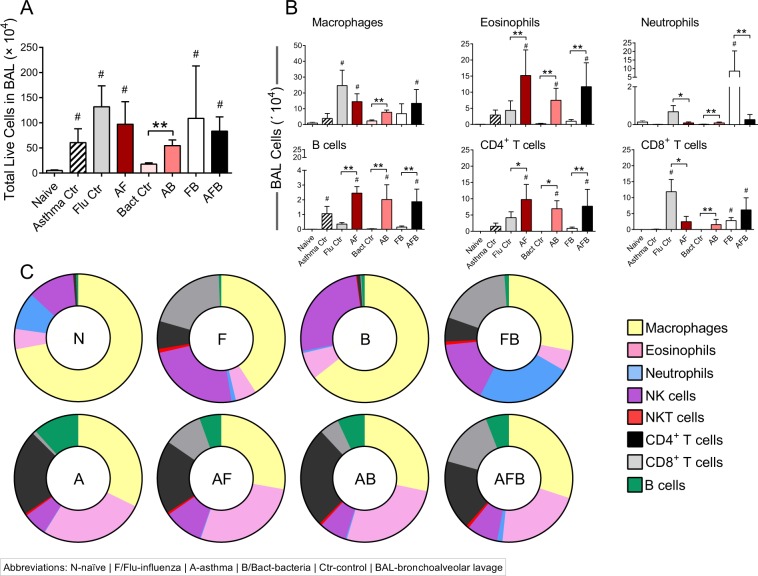
Inflammatory cell profile in the bronchoalveolar lavage (BAL) compartment of mice after triple-disease model. Cells purified from the BAL were enumerated (**A**) and used for flow cytometry to identify cell populations of interest (**B**). The percentage of each cell type was used to normalize data to identify differences in cell types in the airways during various disease conditions (**C**). Data are representative of one study from four independent studies collected at 3 days post *Spn* infection. n = 5–7 mice in each group. Data were analysed by Mann-Whitney test against the direct control group for each experimental condition. *P < 0.05 and **P < 0.01 and by one-way non-parametric ANOVA with Dunn’s multiple comparisons test to determine significant differences from naïve controls (^#^*P* < 0.05).

Inflammation is an important hallmark of both respiratory allergic disease and infections, although dominant cell types differ. We measured the number and types of leukocytes in the airways (bronchoalveolar lavage, BAL) as a marker of disease severity. As expected, inflammatory cells were increased significantly over steady state (naive) after each trigger (Fig. 2A). Macrophages were more abundant in the context of IAV as all groups associated with IAV except the FB group had elevated numbers of macrophages (Fig. 2B). Eosinophils, B cells, and CD4^+^ T cells followed a similar pattern of abundance, wherein cell numbers were significantly higher in co-morbid groups (AF, AB, and AFB) (Fig. 2B). Neutrophil infiltration was markedly higher in the FB group, while CD8^+^ T cell numbers were elevated in the Flu, FB, and AFB groups (Fig. 2B). Animals that were infected only with *Spn*, had very little inflammation and had reduced numbers of all investigated cell types (Fig. 2A,B). Data normalized to individual mouse cell counts and averaged for the group showed the major difference between FB and AFB groups to be granulocyte populations (Fig 2C).

**Figure 3:**
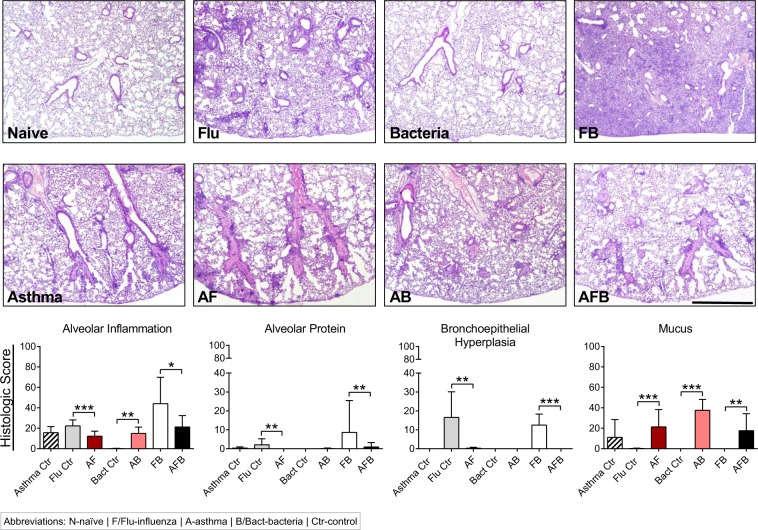
Pulmonary tissue inflammation in triple-disease model. Haematoxylin and eosin stained sections representative of naïve mice and mice in each experimental group (top) were used to quantify parameters of lung damage (bottom). Data are representative of one study from two independent studies collected at 3 days post *Spn* infection. n = 5–7 mice in each group. Data were analysed by Mann-Whitney test against the direct control group for each experimental condition. *P < 0.05, **P < 0.01 and ***P < 0.001.

Over exuberant immune responses are considered a mechanism by which synergistic actions of IAV and *Spn* increased host morbidity and mortality^36^. Analysis of haematoxylin and eosin stained sections of lung tissue showed that widespread parenchymal inflammation was present in IAV-infected mice (Flu, Fig. 3) but that *Spn*-infected mice had minimal areas of inflammation (Bacteria, Fig. 3) most likely due to effective bacterial clearance in otherwise healthy hosts. In contrast, extensive areas of lung parenchyma in FB group mice were consolidated by fluid exudates and inflammatory cells consisting mostly of neutrophils and macrophages (Fig. 3). As expected, inflammation in the asthma-only control mice mostly surrounded the terminal airways (Fig. 3A), and similar inflammatory foci were observed around the small airways in both the AF (Fig. 3) and AB groups (Fig. 3). Significantly, pulmonary lesions in the AFB group mice (Fig. 3) were much less severe than those in the FB group (Fig. 3) and resembled those of AF mice (Fig. 3). Histopathologic scoring of diffuse alveolar damage markers such as alveolar inflammation and protein/fibrin deposition were all much higher in the FB group than in the AFB (Fig. 3), although it is interesting that BAL cell numbers were similar between these groups (Fig. 2A). The higher levels of mucus production in all allergen-exposed mice, irrespective of the presence or type of infectious agent, correlated with the reduced damage and loss of bronchiolar epithelium in these lungs (Fig. 3). These analyses suggest that IAV infection may provide the dominant antigen triggers for the resulting and subsequent inflammation in the lungs.

### Antibiotic treatment impeded the protection from infection-induced morbidity in allergic mice and worsened influenza morbidity while impacting inflammation

**Figure 4:**
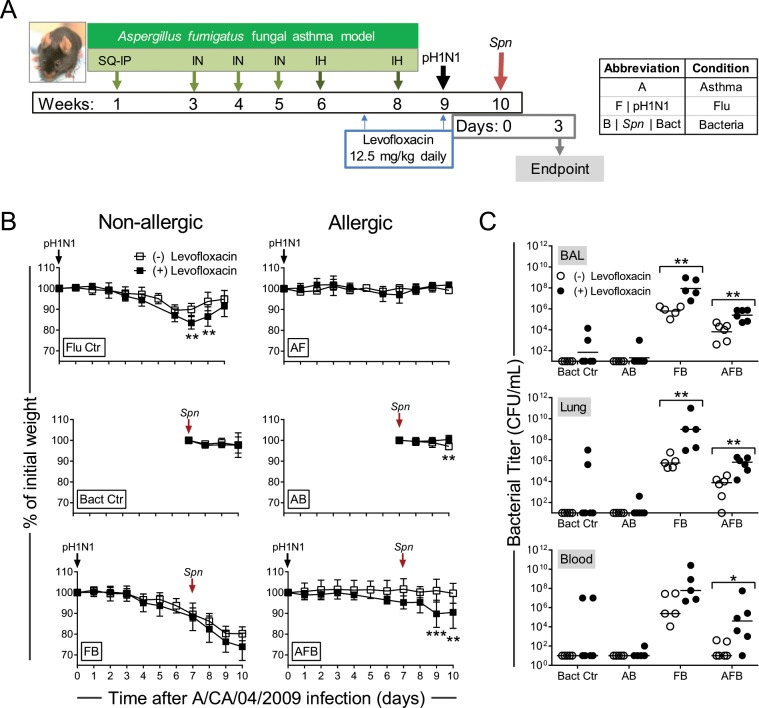
The impact of antibiotic treatment on disease pathogenesis of respiratory infections in asthma. Timeline of model including antibiotic treatments (**A**). Changes in weight in each group in antibiotic treated mice in comparison to mock-treated groups (**B**). Bacterial burden in each disease group in comparison to antibiotic treated groups (**C**). Data are representative of one independent study collected at 3 days post *Spn* infection. n = 5–6 mice per group. Data in B were analysed by two-way ANOVA with Sidak’s multiple comparisons test and Data in C were analysed by Mann-Whitney test. *P < 0.05, **P < 0.01 and ***P < 0.001.

Overuse of antibiotics (Abx)is a growing concern with both short- and long-term implications, many associated with their impact on the gut microbiome^37^. We hypothesized that a microbiome dysbiosis induced by Abx treatment will increase synergistic pathogenesis of IAV and *Spn* in allergic hosts. Mice were treated daily for two weeks with levofloxacin, a commonly used Abx for respiratory infections, to alter the lung microbiome prior to virus infection (Fig. 4A). Antibiotic-treated IAV-infected mice (Flu ctr) had significantly lower nadir than untreated counterparts (Fig. 4B). Abx treatment did not alter weight curves in the other groups except the triple-disease state (AFB), in which allergic co-infected mice treated with  Abx exhibited weight loss resembling the Flu control mice (Fig. 4B). While mice in the Bact Ctr group and AB groups cleared bacteria in all three niches, perhaps at a slightly diminished rate, there were significant differences between the Abx treatment groups that were co-infected where both the FB and AFB groups treated with Abx had elevated levels of *Spn* in all three niches tested (Fig. 4C). Abx treatment made the AFB group mice more susceptible to *Spn*-induced severe morbidity with weight loss and increased bacterial titres (Fig. 4C). These data suggest that protection against respiratory pathogens mediated through the allergic milieu was regulated through the microbiome which is a primary auxiliary effect of antibiotics.

**Figure 5:**
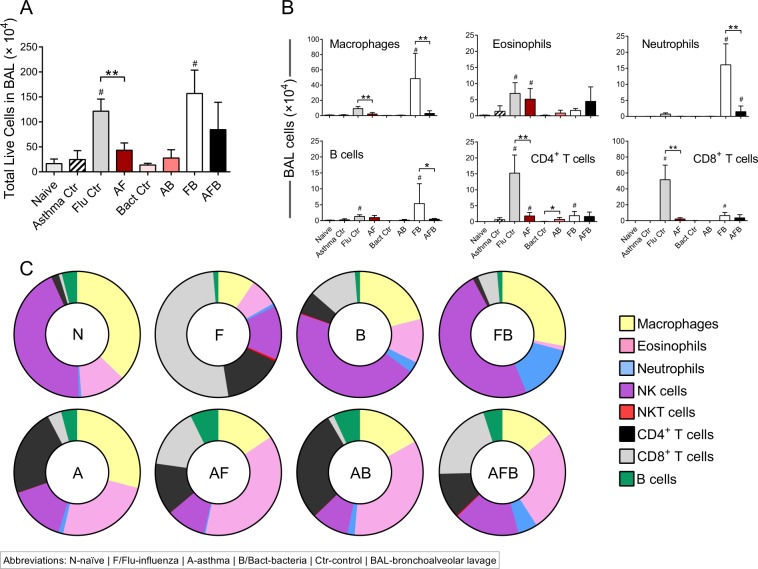
The impact of antibiotic treatment on the immune cell profile in triple-disease model. The number of live leukocytes in the bronchoalveolar lavage (BAL) were enumerated in each group (**A**) and various cell populations were identified by flow cytometry (**B**). Normalized cell populations were used to identify shifts in cell types in each disease state in response to antibiotic treatment (**C**). Data are representative of one study from two independent studies. n = 5–6 mice per group. Data were analysed by Mann-Whitney test against the direct control group for each experimental condition. *P < 0.05 and **P < 0.01 and by one-way non-parametric ANOVA with Dunn’s multiple comparisons test to determine significant differences from naïve controls (^#^*P* < 0.05).

While the total cell numbers were reduced in allergic groups after levofloxacin treatment compared to untreated mice (as shown in Fig. 2 for example), non-allergic mice infected with pathogens had similar cell recruitment patterns (Fig. 5A). Dynamics of cell populations within the airways, however, were altered in all animal groups after Abx treatment. For example, macrophages, neutrophils and B cells were reduce in all groups but increased in the FB group (Fig. 5A). T cells were higher in the Flu control group compared to all other groups and more CD8^+^ T cells were found in the Flu group treated with Abx. Interestingly, eosinophils were reduced in all groups, but most notably in the AB and AFB groups (Fig. 5B). When we normalized the cell types that were measured in each group, the immune profiles were notably of different compositions between groups (Fig. 5C) and with comparison to untreated mice. Macrophage depletion has previously been shown to be an anti-immune strategy used by IAV^38^, and we recently showed that *Cd14* expression increases in response to stimuli, and that its upregulation is more pronounced in the AF group^39^. Since *Cd14* expression correlated with M2 macrophage marker, *Retnla*^39^, it is possible that the reduction in macrophages in the AFB group after Abx treatment inhibited the M2 macrophage phenotype thereby removing an anti-inflammatory/pro-allergic checkpoint^40^.

We investigated the pro-inflammatory cytokine milieu in the BAL and the lungs to better understand the immune pressures influencing infiltrating leukocytes and structural cells (Table S1). In general, the cytokine backdrop in BAL fluid had very little change compared to baseline in all groups except in the FB group (for most analytes) wherein nearly all markers were significantly greater than the AFB group. Similar trends were observed in the lung homogenates. Treatment of mice with Abx affected the cytokine profile in both the BAL and lungs with significant increases occurring in both the FB and AFB groups. However, even after Abx treatment, most cytokines were significantly more abundant in the FB compared to the AFB group.

### Lung mucosal microbiome diversity was reduced by antibiotic treatment

**Figure 6:**
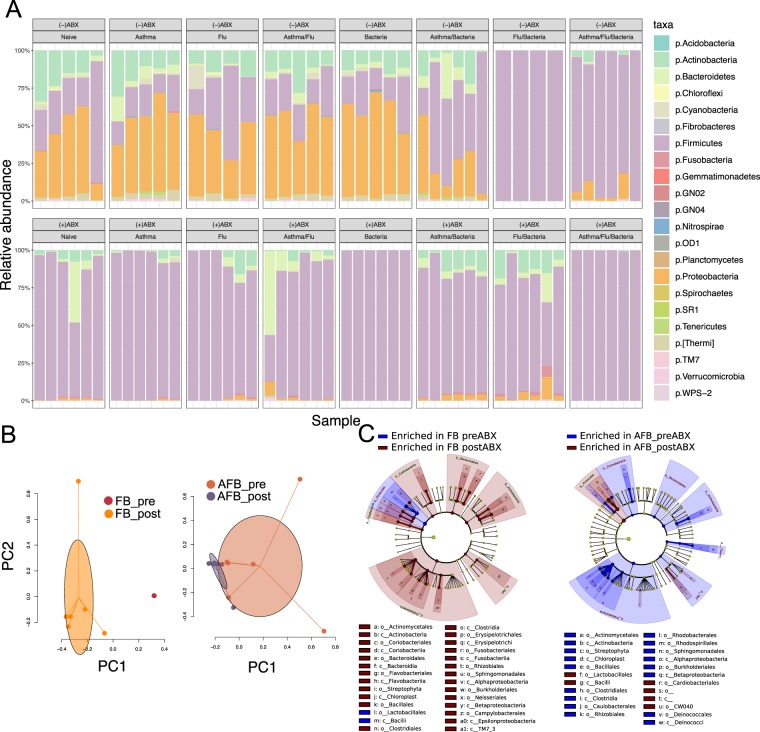
Microbiome profiles of each disease group with and without antibiotics (Abx). The relative proportion of the bacterial taxa identified from the lungs at the phylum level by 16S sequencing for the respective infection groups both with and without antibiotic pre-treatment (**A**). Principle component analysis of the microbial taxa in the Abx-treated mice with influenza-pneumococcal infection or the triple morbidity infection model showing clustering of microbiome for the study groups (**B**). The cladogram for the enriched taxa with and without antibiotic treatment for influenza-pneumococcal co-infection and the impact of allergic asthma on these relative abundances (cutoff is lda >3) (**C**).

Altogether, the altered disease pathogenesis, bacterial burden, and immune profile observed in allergic animals treated with Abx, suggested that Abx could impact the mucosal milieu through the induction of significant changes to the endogenous microbiome that may exist in each disease condition. In order to determine if we were inducing dysbiosis, we analysed the microbiome in the lungs of mice that were treated with Abx in comparison to untreated mice. Differential abundance analyses indicated that the majority of treated mice had reduced taxa abundance. While *Facklamia*, *Bacillaceae*, and *Enterococcus* (p = 0.046) were enriched in BAL of Abx-treated Asthma group, significant depletions were identified in multiple genera especially *Alphaproteobacteria* and *Actinobacteria* (Tables S2, S3). Distinct patterns of taxa identified in the lungs of asthma group (Fig. 6A) showed significant enrichment for *Anaerococcus* of *Clostridia* and *Lactobacillus* in the Abx-treated group, while numerous genera including *Proteobacteria*, *Firmicutes* and *Actinobacteria*, had decreased abundance after Abx treatment. A majority of taxa in the microbiome of Abx-treated Flu control mice were reduced (Fig. 6A) except for a few genera (*Peptoniphilus*, *Selenomonas*, and *Enterococcus*) that were enriched in the BAL samples (data not shown). *Fusobacterium* enriched the lung microbiome of Abx-treated Flu controls while *Streptococcus* enriched the lungs of untreated Flu control mice. All identified taxa in the microbiota of Bacteria-only controls had a significantly reduced abundance after Abx-treatment except for *Streptococcus* that was enriched in the lungs (Fig. 6A). Individual taxa enrichment was observed between Abx-treated and untreated AF group (Fig. 6A), with *Streptococcus* (*P* = 0.03) and *Bradyrhizobium* (*P* = 0.04) enrichment in BAL and *Leptotrichia* in the lung (*P* = 0.04) of treated AF mice. Multiple genera including *Proteobacteria*, *Firmicutes* and *Actinobacteria* were reduced in the BAL of Abx-treated AB mice while *Lactococcus* and *Sphingopyxis* were enriched (data not shown). Similarly separated taxa in the AB group lungs (Fig. 6A) had dynamic enrichment/depletion in *Firmicutes*, *Fusobacteria* and *Proteobacteria* following Abx-treatment.

Clearly separated clusters were evident between the Abx-treated and untreated FB (Fig. 6A,B). While changes in the microbiome were evident in the AFB group after Abx-treatment (Fig. 6A), some overlap still remained as evident by cluster analysis (Fig. 6B). Interestingly, the majority of taxa have increased abundance with *Streptococcus* being the only taxa with decreased abundance after treatment in FB group. Overlapping taxa between Abx-treated and untreated mouse microbiota were only observed in the AFB group (Fig. 6C) wherein most of the identified taxa had reduced abundance; the only exceptions were *Streptococcus* and *Anaerococcus* that were enriched after antibiotic treatment in both niches.

## Discussion

Susceptibility to and severity of respiratory infections are dictated by a multitude of variables from the perspective of both the host and the pathogen. Numerous predisposing conditions have been described to have protective and detrimental roles in the susceptibility of patients to respiratory infections. Such underlying conditions include asthma^41,42^, obesity^43,44^, malnutrition^45^, impaired liver function^46^, and sickle cell disease^47–49^. From the standpoint of respiratory pathogens, pathogenesis tactics for two of the major etiological agents, IAV and *Spn*, are well described in terms of the virulence factors required to successfully infect and cause disease in the respiratory tract^50–54^. Since virulence strategies utilized by pathogens vary considerably based on the host environment, underlying conditions present unique challenges and opportunities for the invading pathogens. The triple-disease murine model developed and utilized here is clinically relevant as asthmatics are considered ‘at risk’ for respiratory infections (both with virus^30^ and bacteria^31,55^) and functions as a tool with which to investigate complex host-pathogen interactions within the allergic host.

Predisposing host conditions provide a unique environment in terms of nutrient bioavailability, receptor expression, and inflammatory status that can alter the virulence strategies of potential pathogens. Influenza synergy with secondary pneumococcal pneumonia is one of the best characterized exacerbations of respiratory infections, with both clinical data and murine models supporting this viral-mediated superinfection^36^. Somewhat counterintuitively, our data show that detrimental synergy between IAV and *Spn*^11,13,26,29,36,42^ is reduced in the context of experimental allergic asthma where IAV + *Spn* co-infection in allergic mice resulted in a dramatically altered inflammatory landscape correlating with less overall lung inflammation and damage, reduced morbidity and enhanced bacterial clearance. Our previous data that allergic mice had swifter viral clearance^24^ raised the possibility that reduced viral load at the time of *Spn* infection may have led to the noted outcomes. Although we were unable to detect infectious virions (data not shown) in any of the IAV-infected groups (Flu ctr, AF, FB, or AFB), the log reduction in viral burden in allergic mice at the time of *Spn* infection could have contributed to decreased morbidity in the AFB group. We did, however, detect the presence of viral genes in the lungs of all animal groups, and since viral *M* gene expression was similar between groups, sustained viral antigens would have had equivalent impact on the hosts. Since H1N1 viral (PR8) rebound after bacterial infection has been previously reported in BALB/c mice^28,35^, albeit not always^56^, synergy between IAV and *Spn* may be differentially regulated in the C57BL/6 strain that is generally more resistant to IAV compared to BALB/c^57^. As such, it is important to consider background strain of mice in studies involving allergens and respiratory infectious agents. Similarly, synergistic interactions between IAV and *Spn* may differ based on the virus strain used in laboratory experiments. It has been previously suggested that a rapidly induced short-lasting inflammatory response may hinder IAV pathogenesis and reduce host pathology^58,59^, and therefore, be beneficial during IAV-*Spn* co-infection^60^. Our data suggest that allergic hosts may hold a similar advantage when infections occur during heightened allergic inflammation in the lungs.

It is becoming increasingly evident that resident bacterial species within the respiratory tract can alter infection susceptibility to exogenous viral and bacterial pathogens^61–64^. Perturbations to the respiratory microbiome may occur during disease states as well as during Abx treatments^65,66^. Early life exposures to microbial agents have been demonstrated both as triggers^67^ and inhibitors^68^ of asthma development based on the agent, atopy, and age^69–71^. Early Abx use correlated with the development of allergic asthma in young children even when accounting for bias inherent from when Abx are commonly prescribed to treat early symptoms of asthma^72,73^. Analysis of microbial communities in the respiratory tract revealed the asthmatic host to have a distinct microbial signature compared to healthy controls, suggesting these alterations in the respiratory microbiome may contribute to the protective capacity of asthma during IAV + *Spn* co-infection. If this mechanism was operative, we hypothesized that perturbation of the microbial communities would diminish the protective capacity of allergic asthma in the context of influenza-pneumococcal co-infection. This was accomplished by Abx administration, which significantly altered the microbial composition of the respiratory tract. Concurrent with this perturbation of microbial communities was a significant decrease in the protective capacity allergic asthma engenders against IAV + *Spn* co-infection. Similar reductions in host protection occur after perturbations to gut microbiome with Abx^74,75^. However, since microbiome disruption with Abx also resulted in a modification to the immune cell profile (with a notable reduction in macrophages and increase in pro-inflammatory cytokines), increased susceptibility to co-infection in the allergic mice may have resulted from a decrease in immunity. While this ‘chicken or egg’ paradox is currently under investigation by our group, it is clear that both the microbiome and immune system play a crucial role in mediating host protection during complicated respiratory infections, and that Abx should be prescribed with caution, especially in patients with chronic underlying conditions as microbiome and immune baselines are likely drifted from an otherwise healthy host.

**Figure 7:**
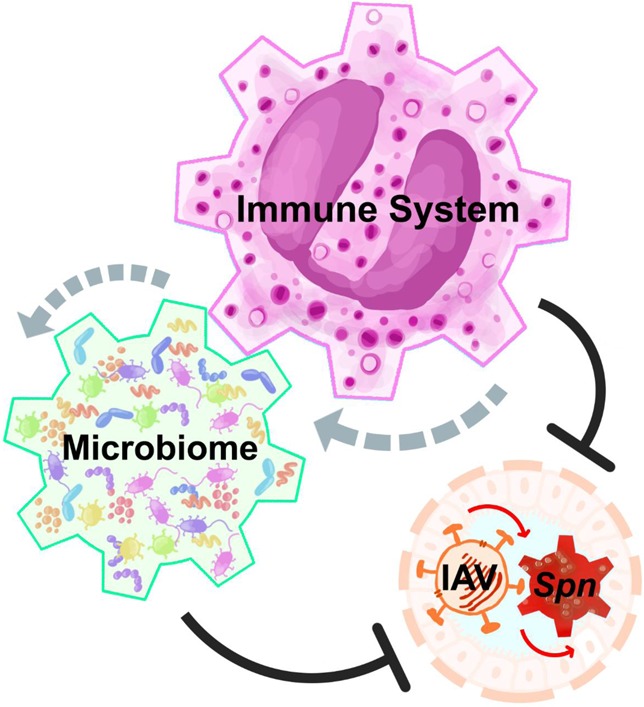
Hypothetical model of host-pathogen interactions during triple-disease condition. Allergen-induced immune responses and endogenous microbiome are altered during co-infection with influenza A virus (IAV) and *Streptococcus pneumoniae* (*Spn*). Each change that occurs in one system will impact the other as systems modulate one another. We propose that the allergic milieu hinders pathogen synergy thereby protecting mice with asthma from severe morbidity and mortality associated with IAV and *Spn* co-infection.

The extensive alterations in the respiratory tract during allergic asthma encompass both immunological and microbiological differences that can have a profound impact on susceptibility to infection. These data suggest that allergic asthma may provide a significant advantage in terms of morbidity and mortality against certain respiratory infectious agents, and that this protection is partly mediated by alterations in the resident flora and immune milieu that may be interrelated and interdependent (Fig. 7). Due to the complex interplay between the microbiota and host immunity, there are likely additional host signaling pathways that modulate allergic host sensitivity to infection, leading to the inhibition of viral-bacterial synergy or discordance (Fig. 7). The observation that Abx partially ameliorated this protection also underscores the importance of appropriate Abx use, as prior Abx exposure enhances the susceptibility of mice with allergic asthma to subsequent infection. This is of particular importance to asthmatic patients, as the early symptoms of asthma are similar to respiratory infection, potentially resulting in inappropriate Abx usage^76^. In this study, we utilized H1N1 influenza virus isolate from the 2009 ‘Swine Flu’ pandemic with *Spn* due to the increased vulnerability to *Spn* similar to trends seen during the 1918 ‘Spanish Flu’ pandemic^77,78^. Similar associations have been noted to occur during some H2N2 and H3N2 seasons, although very few reports address possible synergistic mechanisms between influenza B virus and bacteria^36,79–81^. Therefore, it would be of interest to utilize this animal model system to determine possible allergic host-pathogen-microbiome interactions that may be operative with different influenza virus subtypes in combination with both Gram positive and negative strains of bacteria.

Asthma is a complicated syndrome that develops through intricate gene:environment interaction. As a result, numerous endotypes and subtypes exist complicating disease investigation and treatment^82^. This heterogeneity in asthmatics could explain why not all patients with underlying asthma were hospitalized during the 2009 influenza pandemic, and could also explain the disease course and outcome variability observed in the hospitalized asthmatics^17^. Our previous findings emphasize that the timing of IAV infection and the state of the allergic milieu at the time of infection impact the outcome of influenza disease in mice with underlying asthma^24^. Our more recent discovery that heightened eosinophilia during the IAV-induced asthma exacerbation works to protect the host from severe influenza morbidity^25^ further reveals that mediators in the allergic milieu plays a significant role in anti-influenza responses. Congruently, data provided in this study underscore the complex and interwoven relationship between host immune status and the resident bacterial flora in terms of infection susceptibility. Therefore, it is important to determine parameters that can be measured in asthmatics that can give insight into possible mechanisms that may be at play in the lungs (inaccessible in the clinical setting) during respiratory infections in order to understand how each asthmatic may respond. Individual patient responses to the invading pathogen will be dependent on the endotype and subtype of asthma, type of infection, other underlying diseases (obesity, diabetes, hypertension, etc.), age/chronicity of asthma (whether lung remodeling may have occurred), among others. Animal models that can recapitulate the nuances of these patients are crucial to gain knowledge into immune and structural parameters that surround this obfuscated clinical setting and underscores the importance of systems biology in delineating host-pathogen interactions in complex circumstances as in hosts with underlying chronic disease.

## Materials and Methods

### Ethics statement

All work with infectious agents described herein were done in accordance with protocols approved by the Institutional Biosafety Committees at St. Jude Children's Research Hospital (SJCRH) and the University of Tennessee Health Science Center (UTHSC). All animal work described herein were performed in strict accordance with the Institutional Animal Care and Use Committees at SJCRH and UTHSC.

### Pathogen strains and growth

A clinical Influenza A Virus isolate recovered during the 2009 influenza A virus pandemic (A/CA/04/2009) gifted by Dr. Webby (SJCRH), was propagated in Madin-Darby canine kidney (MDCK) cells, sequence verified to be void of mutations in HA and NA genes, and stored as single use aliquots at −80 °C. The concentration of virus was determined by using the TCID_50_ method in MDCK cells and virus was diluted in sterile PBS to desired concentration for mouse inoculations.

*S. pneumoniae* A66.1 L is a type 3 strain constitutively expressing luciferase (Francis KP *et al*., 2001), gifted by Dr. Jon McCullers (UTHSC) was cultured in Todd-Hewitt broth (Difco Laboratories, Detroit, MI) supplemented with yeast extract (ACROS Organics, NJ) to OD_620_ 0.15 corresponding to log growth phase. Cultures were centrifuged at 2671 *g*/4 °C, re-suspended in fresh Todd-Hewitt Yeast broth supplemented with 30% autoclaved glycerol, then frozen in single use aliquots at −80 °C with concentration determined by enumerating colony growth on agar plates with 5% sheep blood (Remel, Thermo Fisher, Lenexa, KS) at the time of freezing, and confirmed at least one week after storage. To prepare mouse inocula, A66.1 L was thawed at room temperature and serially diluted in PBS in 15 mL tubes, then vortexed. Inocula concentration was confirmed by colony growth on blood agar plates.

### Animals and housing conditions

Since no discernible differences in gender were noted after subjecting mice to the fungal asthma and influenza model, female mice were used in these studies. Six week-old female C57BL/6J mice were purchased from Jackson Laboratories (Bar Harbor, ME) and maintained in sterile microisolator cages on α-dri bedding within the animal biosafety level-2 facilities at SJCRH and University of Tennessee Health Science Center for 1 week prior to being used in experiments. The animal housing facilities were on a 12 h light-dark cycle and all work with animals was done during the light cycle. Animals were fed autoclaved chow and provided autoclaved water in bottles *ad libitum*.

### Mouse fungal asthma model

Mice were subjected a previously described and characterized model of *Aspergillus fumigatus*-induced allergic asthma^32,83^. In brief, whole *A. fumigatus* extract (Greer Labs, Lenoir, NC) was used to sensitize mice over a period of five weeks prior first by subcutaneous (SC) and intraperitoneal (IP) injections with Alum (Thermo Fisher, Waltham, MA) followed by intranasal (IN) delivery of antigen at the depicted timepoints in Fig. 1A. One week after the third IN exposure, mice were anesthetized with a ketamine and xylazine cocktail and exposed to airborne dry fungal conidia in a nose-only inhalation (IH) chamber for 10-minutes two weeks apart (Fig. 1A)^27^. Although challenged with live fungal conidia which can be located within the lungs^83^, fungal growth is inhibited in immunocompetent mice. Allergen sensitized and challenged mice were referred to as the “Asthma Ctr” group. The authors note that the term “asthma” is not ideal for characteristics induced experimentally in mice, however, we do so for ease of reference and understanding in readers that may not be well versed on the nuances of using mice for asthma research.

### Animal infections

Mice were lightly anesthetized with isoflurane and intranasally infected with 1000 TCID_50_ A/CA/04/2009, diluted in 50 µL PBS. The infection dose was selected to induce morbidity but not mortality in mice^24^. Seven days after the viral infection, mice were anesthetized with isoflurane and intranasally infected with 600 colony forming units (CFU) of A66.1 L in 100 µL PBS. This dose was selected as one that could be quickly cleared by healthy mice, and yet cause severe disease in mice recovering from influenza. Each mouse was weighed prior to challenge and every 24 hours for 6 days after the *Spn* infection to monitor weight change. Animals that lost more than 30% of their starting body weight, or exhibited severe signs of morbidity, were euthanized for ethical reasons and recorded as having died on that day. Each group of mice that were infected with a single agent were named after the pathogen as influenza virus (Flu Ctr) and bacteria (Bact Ctr) only. Naïve (N) mice that had no treatments served to determine baseline immune and microbiome information. Allergic mice that were infected with influenza (Flu) virus^24^ were referred to as the “Asthma and Influenza” (AF) group and those additionally infected with bacteria were considered the “Asthma, Influenza, and Bacteria” (AFB) group. Allergic mice that were infected with bacteria were referred to as the “Asthma and Bacteria” (AB) group.

### Antibiotic treatments

Naïve and mice in the various treatment groups were intraperitoneally administered 12.5 mg/kg levofloxacin (Akorn Inc., Lake Forest, IL) daily at the equivalence of one week after the first allergen challenge and ending on the day of viral infection. No visible differences occurred during disease pathogenesis in mice after antibiotic treatment.

### Bioluminescence measurements

The use of the A66.1 L strain afforded the visualization of bacteria during active infection. We measured bioluminescence by a Lumina IVIS CCD camera (Perkin Elmer, Waltham, MA). Images were processed with Living Image software, version 4.5.5. Settings for image acquisition were a one second photograph followed by one-minute luminescence measurement with the bining set at four.

### Tissue harvest and pathogen enumeration

Tissues were harvested within a class 2 A biosafety cabinet with strict adherence to aseptic technique to ensure that samples were not contaminated by exposure to environmental agents. Bronchoalveolar lavage (BAL) was performed using two consecutive infusions of 1 mL sterile PBS. BAL cells were cytospun and stained with Diff-quik (StatLab, McKinney, TX) for visualization. Cells were, then centrifuged at 4 °C and BAL fluid (BALF) was stored at −80 °C until use. Red blood cells in the cell pellet were removed by lysis with cold water and resultant cells enumerated on a Countess Automated Cell Counter (Invitrogen, Carlsbad, CA) then stained for flow cytometry.

Blood was recovered from the chest cavity, and serum was separated by centrifugation and stored at −80 °C. Lungs were excised and homogenized in 1 mL PBS containing cOmplete Proteinase Inhibitor Cocktail (Roche Diagnostics, Mannheim, Germany) then centrifuged at 4 °C to remove cell debris. Cell-free supernatant was stored at −80 °C until cytokine quantitation. In some experiments, spleen, mediastinal lymph nodes and left lung lobes were perfused with 10% neutral-buffered formalin and processed for hematoxylin and eosin staining.

Whole lung homogenate, whole blood, and BAL samples were serially diluted in sterile PBS and 10 µL of dilutions were plated on blood agar plates. Plates were incubated at 37 °C with 5% CO_2_ for 12–13 hours, and A66.1L colony growth was enumerated.

Serially diluted lung homogenates were added to confluent MDCK cells and incubated for 1 hour at 37 °C with 5% CO_2_. Cells were washed twice to remove any unadsorbed virus and incubated for 72 hours in media containing 1 µg/mL TPCK-trypsin (Worthington Biochemicals, Lakewood, NJ) at 37 °C with 5% CO_2_. The amount of IAV in the supernatant was determined by hemagglutination of chicken red blood cells and calculated by the Reed Muench method. Extracted RNA was used in conjunction with the Simplexa™ Flu A/B kit from Focus Diagnostics (Cypress, CA) to determine the expression of viral *M* gene in each sample.

### Histopathologic analysis

Lungs were first infused and then immersion-fixed in 10% neutral buffered formalin before processing and embedding in paraffin. Tissue sections were stained with hematoxylin and eosin, and the severity and extent of specific pulmonary lesions such as interstitial and alveolar inflammation, alveolar protein exudate, hyaline membrane formation, septal thickening, epithelial hypeplasia, and denuded bronchioles, were assessed and graded in a blinded manner by a veterinary pathologist. Separate severity grades for each type of lesion were assigned as follows: 0, no lesions detected; 1, minimal, rare, barely detectable lesions; 2, mild multifocal, small focal, or widely separated lesions; 3, moderate, multifocal, and prominent lesions; 4, marked, extensive-to-coalescing areas; and 5, severe and extensive lesions with pulmonary consolidation. These severity grades were then converted to weighted semi-quantitative scores as follows: 0 = 0; 1 = 1; 2 = 15; 3 = 40; 4 = 80; and 5 = 100.

### Flow cytometric analysis

Cells in the airways were identified using flow cytometry. BAL cells were incubated with human γglobulin to prevent nonspecific binding of antibodies to Fc-receptor, then stained with fluorescently tagged antibodies. Samples were fixed with BD Biosciences stabilizing fixative and stored in the dark at 4 °C until acquisition on a LSR Fortessa (BD Biosciences, San Jose, CA). Data were analyzed using FlowJo v 10.1r5 (FlowJo LLC, Ashland, OR). Unstained cells, single-color controls and isotype controls were used for instrument settings and compensation. Antibodies were purchased from BD Biosciences unless specified otherwise. Antibodies used in this study include: CD19-PerCP/Cy5.5 (1D3), CCR3-A647 (83103), CD3e-PE/Cy7 (145-2C11), CD4-A700 (RM4-5), CD8a-FITC (53–6.7), Ly6G-V450 (1A8), Mac3-Biotin (ebioABL-93, eBioscience|Thermo Fisher, Waltham, MA), NK1.1-APC/Cy7 (PK136), NP-dextramer-PE (Immudex, Fairfax, VA), Siglec-F-PE-CF594 (E50-2440), Streptavidin-BV605 (BioLegend) and matching isotypes control antibodies. Gating strategy employed to identify cell populations is provided as Fig. S1.

### Cytokine quantitation

Cytokines present in the airways and interstitial lung tissue were quantified using magnetic Luminex multiplex assays (R&D) according to the manufacturer’s instructions. BALF and cell-free lung homogenates were stored at −80 °C in single-use aliquots after harvest until cytokine assay, and used undiluted or diluted ½ in Calibrator Diluent (R&D Systems (Minneapolis, MN), respectively. Beads were read using a Luminex MagPix (R&D Systems). Analyte concentration was determined using MagPix software xPONENT 4.2. Samples which had a reading below the limit of detection were assigned the value of the limit of detection. The mean concentration for the group for each analyte was used to calculate the fold change compared to the mean concentration of the same analyte in the naïve group.

### 16S sequence analysis

Whole lung homogenates and BAL were centrifuged at 500 × *g* at 4 °C to remove cell debris, then subsequently centrifuged at 2671 × *g* at 4 °C to pellet bacteria in the samples. Bacterial pellets were re-suspended in 50 µL of BALF or lung homogenate supernatant and stored at −80 °C until DNA extraction. Samples were thawed on ice and DNA was extracted using FastDNA-96 Soil Microbe DNA kit (MP Biomedicals, Santa Ana, CA) according to the manufacturer’s instructions, and a FastPrep-96 instrument (MP Biomedicals). Extracted nucleic acid was stored at −80 °C until use.

The V1–V3 variable region of the bacterial 16S gene was amplified using the Bioo NEXTflex 16S amplicon library preparation kit. Recently, Dickson *et al*.^84^, demonstrated that a microbial signature, albeit small, was noted in sterile procedural/reagent specimens used to harvest samples in mice^84^. Also, due to variation in contamination of extraction kits and reagents, establishing the baseline contamination for microbial contamination is of importance when analyzing low-abundance microbial communities. Although we did not subject reagent controls through our pipeline, we selected amplification cycles based on qRT-PCR of mock-extracted samples to ensure minimal contamination effects from extraction protocol or reagents. The multiplexed products were then subsequently utilized for high-depth sequencing on the Illumina Mi-seq platform to obtain comprehensive relative abundance of the bacterial composition at the respective time points with 300 bp paired end read lengths.

The quality of the raw 16S rRNA pair-ended reads are initially examined by FastQC^85^. Low quality reads and bases are trimmed by Trim Galore^86^. The reads are then merged by PANDAseq^87^. and subsequently processed by QIIME^88^. The open reference mapping protocol of QIIME was used for OTU assignment. Specifically, the read sequences were clustered into Operational Taxonomic Units (OTUs) at 97% sequence similarity using the UCLUST algorithm^89^. A representative sequence was then selected from each OTU for taxonomic assignment using the Greengenes database^90^ as the reference. The sequences failed to hit the reference database were subsampled and *de-novo* re-clustered; the cluster centroid was used as new reference. These *de-novo* new references were used to re-interrogate all of the reads that failed to hit the reference and assign them to an OTU. A final round of *de novo* OTU picking was performed for those reads that still failed to hit any reference. Finally, taxonomy was assigned to the representative sequence of each OTU. The final result contained an OTU matrix detailing the information about the number of sequences in each OTUs and their taxonomy assignment.

The alpha diversity estimates were calculated using the R Phyloseq package^91^. Kruskal-Wallis non-parametric tests was used to test the significance of the diversity difference. The linear discriminant analysis (LDA) effect size (LEfSe) method was used to test the significant difference of relative abundance of taxa among groups^92^. The LEfSe tool first performed non-parametric factorial Kruskal-Wallis (KW) sum-rank test to identify features with significant differential abundance with respect to the class of interest; a set of subsequent pairwise tests among subclasses was performed using the Wilcoxon rank-sum test. LEfSe then estimated the effect size of each differentially abundant feature to perform dimension reduction by LDA.

### Study design and statistical analyses

All stated experiments were independently performed as a ‘study’ with 5–7 mice in each treatment group utilizing at least 140 mice for each study for all timepoints and the study was repeated three times in order to maintain high scientific rigor and ensure reproducibility of the findings. The microbiome studies were independently performed two times. Data are represented from one independent study and graphed using GraphPad Prism v.6.01 which was also used to perform the statistical analyses. Statistical tests used for each dataset is stated in the Figure legends.

## Supplementary information

Supplemental Information

Supplemental Table 2

Supplemental Table 3

## Data Availability

The datasets generated and/or analysed during the current study are available in the NCBI BioProject repository under Accession Code PRJNA530763. Data were previously presented in part at the American Association of Immunologists annual conference (2013 Honolulu, HI and 2016 Seattle, WA) and the American Thoracic Society annual conference (2018 San Diego, CA and 2019 Dallas, TX).
