# Supplementary material for: Allergic inflammation alters the lung microbiome and hinders synergistic co-infection with H1N1 influenza virus and Streptococcus pneumoniae in C57BL/6 mice
Source: Sci Rep. 2019 Dec 18;9:19360. doi: 10.1038/s41598-019-55712-8 (PMC6920369; doi:10.1038/s41598-019-55712-8)
Supplement: Supplementary file 1 — Supplemental Information [file 41598_2019_55712_MOESM1_ESM.docx]

**SUPPLEMENTARY INFORMATION**

**Allergic inflammation alters the lung microbiome and hinders synergistic co-infection with H1N1 influenza virus and *Streptococcus pneumoniae* in C57BL/6 mice**

Kim S. LeMessurier,^1,2,#^ Amy R. Iverson,^3,#^ Ti-Cheng Chang,^4^ Maneesha Palipane,^1,2^ Peter Vogel,^5^ Jason W. Rosch,^6,¶^ and Amali E. Samarasinghe^1,¶,*^

^1^Department of Paediatrics, College of Medicine, University of Tennessee Health Science Center, Memphis, TN 38103; ^2^Children’s Foundation Research Institute, Memphis, TN 38103; Departments of ^4^Computational Biology, ^5^Veterinary Pathology, and ^6^Infectious Diseases at St. Jude Children’s Research Hospital, Memphis, TN 38105.

^#^ and ^¶^ indicate equal contributions by authors. * indicates corresponding author.

**Figure S1:** Gating strategy used to identify leukocyte populations in the brochoalveolar lavage cell contents in each mouse.

**Table S1. Mucosal Cytokines in Mouse Bronchoalveolar Lavage Fluid (BALF) and Lung Homogenate.**

*Untreated mice*

| **BALF** | **Fold increase over Naïve** | | | | | | |
| --- | --- | --- | --- | --- | --- | --- | --- |
|  | **Asthma** | **Flu-ctr** | **Bact-ctr** | **AF** | **AB** | **FB** | **AFB** |
| CRG2 | 1.00 | 1.00 | 1.00 | 1.00 | 1.00 | 10.75^+^ | 1.86^*^ |
| G-CSF | 1.00 | 1.00 | 1.00 | 1.00 | 1.00 | 14.19^+^ | 1.58^*^ |
| IFNγ | 1.00 | 1.00 | 1.00 | 1.00 | 1.00 | 3.89^+^ | 1.03^*^ |
| IL-1α | 1.00 | 1.00 | 1.00 | 1.00 | 1.00 | 1.49 | 1.00 |
| IL-6 | 1.00 | 1.04 | 1.00 | 1.00 | 1.00 | 23.66^+^ | 5.68^*^ |
| IL-10 | 1.00 | 1.00 | 1.00 | 1.00 | 1.00 | 1.00 | 1.00 |
| KC | 1.00 | 1.23 | 1.00 | 1.00 | 1.00 | 5.10^+^ | 1.38^*^ |
| M-CSF | 1.00 | 1.00 | 1.00 | 1.00 | 1.00 | 1.25 | 1.00 |
| MCP-1 | 1.00 | 1.00 | 1.00 | 1.00 | 1.00 | 9.33^+^ | 1.15^*^ |
| MIP-1α | 1.00 | 1.00 | 1.00 | 1.00 | 1.00 | 18.89^+^ | 0.55^*^ |
| MIP-1β | 1.00 | 1.00 | 1.00 | 1.00 | 1.00 | 78.25^+^ | 11.57^*^ |
| MIP-2 | 1.00 | 1.02 | 1.00 | 1.00 | 1.00 | 54.25^+^ | 4.40^*^ |
| RANTES | 1.00 | 1.54 | 1.00 | 1.00 | 1.00 | 10.50^+^ | 1.61^*^ |
| TNFα | 1.00 | 1.00 | 1.00 | 1.00 | 1.00 | 26.33^+^ | 3.88^*^ |

*Levofloxacin-treated mice*

| **BALF** | **Fold increase over Naïve** | | | | | | |
| --- | --- | --- | --- | --- | --- | --- | --- |
|  | **Asthma** | **Flu-ctr** | **Bact-ctr** | **AF** | **AB** | **FB** | **AFB** |
| CRG2 | 1.00 | 1.00 | 1.00 | 1.00 | 1.00 | 11.85^+^ | 9.39^+#^ |
| G-CSF | 1.00 | 1.00 | 1.00 | 1.00 | 1.00 | 34.05^+^ | 7.48^*#^ |
| IFNγ | 1.00 | 1.00 | 1.00 | 1.00 | 1.00 | 2.26^+^ | 1.57^#^ |
| IL-1α | 1.00 | 1.00 | 1.00 | 1.00 | 1.00 | 2.74^+#^ | 1.00^*^ |
| IL-6 | 1.00 | 1.74 | 1.00 | 1.00 | 1.00 | 28.34^+^ | 19.97^+#^ |
| IL-10 | 1.00 | 1.00 | 1.00 | 1.00 | 1.00 | 13.07^+#^ | 1.00^*^ |
| KC | 1.00 | 1.14 | 1.00 | 1.00 | 1.00 | 5.67^+^ | 4.47^+#^ |
| M-CSF | 1.00 | 1.00 | 1.00 | 1.00 | 1.00 | 1.58^+^ | 1.00^*^ |
| MCP-1 | 1.01 | 1.00 | 1.00 | 1.00 | 1.00 | 11.84^+#^ | 4.85^+#*^ |
| MIP-1α | 1.82 | 1.10 | 1.00 | 1.00 | 1.00 | 85.99^+#^ | 6.53^*^ |
| MIP-1β | 1.57 | 1.00 | 1.00 | 1.00 | 1.00 | 83.89^+^ | 48.14^+*#^ |
| MIP-2 | 7.18 | 2.07 | 1.00 | 2.10 | 1.33 | 144.52^+#^ | 19.94^*^ |
| RANTES | 1.00 | 2.14 | 1.13 | 1.32 | 1.00 | 9.91^+*^ | 4.78^+*#^ |
| TNFα | 1.00 | 1.47 | 1.00 | 1.00 | 1.00 | 24.03^+^ | 17.41^+*#^ |

^+^ p<0.05 compared to Naïve

* p<0.05 AFB compared to FB

^#^ p<0.05 levofloxacin-treated compared to untreated

*Untreated mice*

| **Lung tissue** | **Fold increase over Naïve** | | | | | | |
| --- | --- | --- | --- | --- | --- | --- | --- |
|  | **Asthma** | **Flu-ctr** | **Bact-ctr** | **AF** | **AB** | **FB** | **AFB** |
| CRG2 | 1.00 | 1.52 | 1.00 | 1.00 | 1.00 | 36.30^+^ | 3.23^*^ |
| G-CSF | 1.00 | 1.00 | 1.00 | 1.00 | 1.00 | 55.24^+^ | 3.19^*^ |
| IFNγ | 0.89 | 1.08 | 1.08 | 0.98 | 1.21 | 7.12^+^ | 2.38^*^ |
| IL-1α | 1.04 | 0.70 | 1.15 | 0.84 | 1.12 | 9.67 | 1.78^*^ |
| IL-6 | 0.99 | 0.55 | 1.02 | 0.74 | 0.87 | 11.64 | 2.75 |
| IL-10 | 1.00 | 0.65 | 1.08 | 0.92 | 1.06 | 1.06 | 1.13 |
| KC | 1.06 | 0.56 | 1.11 | 0.82 | 0.95 | 2.07 | 1.1 |
| M-CSF | 0.86 | 0.74 | 1.05 | 0.88 | 0.96 | 2.27 | 0.83 |
| MCP-1 | 1.00 | 3.11 | 1.00 | 1.29 | 1.21 | 38.58^+^ | 3.05^*^ |
| MIP-1α | 1.88 | 7.11 | 1.00 | 2.71 | 3.13 | 185.27^+^ | 10.79^*^ |
| MIP-1β | 1.00 | 1.00 | 1.00 | 1.00 | 1.00 | 209.57 | 18.17 |
| MIP-2 | 1.75 | 4.13 | 1.11 | 2.01 | 2.45 | 994.52^+^ | 65.47^*^ |
| RANTES | 1.42 | 3.87 | 1.00 | 2.22 | 1.50 | 19.24^+^ | 3.03^*^ |
| TNFα | 1.01 | 1.20 | 1.14 | 1.16 | 1.19 | 24.78^+^ | 3.09^*^ |

*Levofloxacin-treated mice*

| **Lung tissue** | **Fold increase over Naïve** | | | | | | |
| --- | --- | --- | --- | --- | --- | --- | --- |
|  | **Asthma** | **Flu-ctr** | **Bact-ctr** | **AF** | **AB** | **FB** | **AFB** |
| CRG2 | 1.00 | 1.46 | 1.00 | 1.00 | 1.00 | 28.46^+^ | 41.95^+*#^ |
| G-CSF | 1.00 | 1.00 | 2.32 | 1.00 | 1.00 | OHR | 24.60^+#^ |
| IFNγ | 1.00 | 1.00 | 1.00 | 1.00 | 1.33 | 3.41^#^ | 9.21^+*#^ |
| IL-1α | 1.54 | 1.08 | 1.34 | 1.53 | 2.92 | 25.9^+#^ | 24.55^+#^ |
| IL-6 | 1.44 | 0.96 | 1.40 | 1.39 | 2.70 | 457.70^+#^ | 42.32^*^ |
| IL-10 | 1.17 | 0.95 | 1.00 | 1.03 | 2.23 | 46.6^+#^ | 1.9^*^ |
| KC | 1.22 | 0.67 | 1.27 | 1.11 | 2.69 | 4.75^+#^ | 4.02^+#^ |
| M-CSF | 1.00 | 1.00 | 1.00 | 1.00 | 1.16 | 76.80^+#^ | 1.64^*^ |
| MCP-1 | 1.00 | 2.14 | 0.86 | 1.49 | 1.00 | 66.07^+#^ | 17.55^*^ |
| MIP-1α | 1.02 | 5.32 | 1.00 | 2.03 | 1.03 | OHR | 75.72^+#^ |
| MIP-1β | 1.08 | 1.00 | 1.00 | 1.00 | 1.00 | 978.33^+#^ | 107.51^+*^ |
| MIP-2 | 6.72 | 3.50 | 3.25 | 2.28 | 2.28 | OHR | 576.50^#^ |
| RANTES | 1.83 | 6.87 | 1.28 | 5.11 | 2.75 | 18.47^+^ | 24.60^+*#^ |
| TNFα | 1.03 | 1.00 | 1.00 | 1.00 | 1.68 | 27.42^+^ | 17.48^+#^ |

^+^ p<0.05 compared to Naïve

* p<0.05 AFB compared to FB

^#^ p<0.05 levofloxacin-treated compared to untreated
